# Spatiotemporal interactions between wild boar and cattle: implications for cross-species disease transmission

**DOI:** 10.1186/s13567-014-0122-7

**Published:** 2014-12-12

**Authors:** Jose A Barasona, M Cecilia Latham, Pelayo Acevedo, Jose A Armenteros, A David M Latham, Christian Gortazar, Francisco Carro, Ramon C Soriguer, Joaquin Vicente

**Affiliations:** SaBio (Health and Biotechnology), IREC, National Wildlife Research Institute (CSIC-UCLM-JCCM), Ciudad Real, Spain; Landcare Research, PO Box 69040, Lincoln, Canterbury 7640 New Zealand; Estación Biológica de Doñana, Consejo Superior de Investigaciones Científicas (CSIC), Sevilla, Spain

## Abstract

Controlling infectious diseases at the wildlife/livestock interface is often difficult because the ecological processes driving transmission between wildlife reservoirs and sympatric livestock populations are poorly understood. Thus, assessing how animals use their environment and how this affects interspecific interactions is an important factor in determining the local risk for disease transmission and maintenance. We used data from concurrently monitored GPS-collared domestic cattle and wild boar (*Sus scrofa*) to assess spatiotemporal interactions and associated implications for bovine tuberculosis (TB) transmission in a complex ecological and epidemiological system, Doñana National Park (DNP, South Spain). We found that fine-scale spatial overlap of cattle and wild boar was seasonally high in some habitats. In general, spatial interactions between the two species were highest in the marsh-shrub ecotone and at permanent water sources, whereas shrub-woodlands and seasonal grass-marshlands were areas with lower predicted relative interactions. Wild boar and cattle generally used different resources during winter and spring in DNP. Conversely, limited differences in resource selection during summer and autumn, when food and water availability were limiting, resulted in negligible spatial segregation and thus probably high encounter rates. The spatial gradient in potential overlap between the two species across DNP corresponded well with the spatial variation in the observed incidence of TB in cattle and prevalence of TB in wild boar. We suggest that the marsh-shrub ecotone and permanent water sources act as important points of TB transmission in our system, particularly during summer and autumn. Targeted management actions are suggested to reduce potential interactions between cattle and wild boar in order to prevent disease transmission and design effective control strategies.

**Electronic supplementary material:**

The online version of this article (doi:10.1186/s13567-014-0122-7) contains supplementary material, which is available to authorized users.

## Introduction

Most pathogens of concern to livestock are able to cross-infect multiple host species, including wildlife, and therefore in areas where wildlife and livestock co-occur (i.e. interface areas), pathogens can emerge and establish in these sympatric host populations [1]. For example, foot and mouth disease, rabies, anthrax, brucellosis and bovine tuberculosis (TB) have all been shown to be reciprocally transmissible between livestock and wildlife [2–6]. In this context, the demography and behaviour of the hosts’ populations can play an important role in intra- and interspecific pathogen transmission by determining contact rates and environmental exposure [7]. If resources that are commonly used by both domestic and wild species are aggregated, this can result in high spatial and/or temporal overlap between two or more species [6–9], further increasing the probability of disease transmission. How habitat use by hosts affects direct and indirect interactions among hosts is fundamental in understanding multi-host disease transmission [5], and is critical for designing scientifically-based disease control strategies [10]. Nonetheless, the role that spatial and temporal interactions between livestock and wildlife play in exposure to pathogens and disease transmission remains mostly unknown [11,12].

Tuberculosis caused by the *Mycobacterium tuberculosis* complex is an important re-emerging zoonotic disease shared between domestic cattle and wildlife, and the control of this disease is largely limited by the existence of wildlife reservoirs [13–15]. In the United Kingdom, for instance, cattle may become infected with TB by using farm buildings (feed stores and cattle sheds) and grazing on grass that has been contaminated with urine, faeces, sputum or wound exudates of badgers (*Meles meles*) [16,17]. In the United States, white-tailed deer (*Odocoileus virginianus*) and cattle often share rangeland resources, including water sources and feeders, although temporal segregation between these species is often observed [9]. In the Iberian Peninsula, wild boar (*Sus scrofa*) are the main wild maintenance host of TB [18]. Recent studies from Spain suggest that TB infection can spread not only by direct contact among individuals but also by indirect transmission [19], with water sources being high risk areas where pathogen transmission can occur between wildlife and cattle through consumption of short-term infected water [20]. However, epidemiological studies at the interface between livestock and important disease-carrying wildlife, such as wild boar, remain scarce.

Despite compulsory testing and culling of infected cattle, TB infection rates in cattle populations are persistently high in Doñana National Park (DNP), southern Spain [21]. The TB-host community of DNP includes wild boar, red deer (*Cervus elaphus*) and fallow deer (*Dama dama*), all of which occur sympatrically in areas used for traditional cattle husbandry. Interestingly, the populations of these three wildlife hosts exhibit common spatial patterns of TB infection across DNP, which may be explained by resource use and behaviour of these species [21,22]. Recent advances in global positioning system (GPS) technology for monitoring wildlife has proven useful for assessing fine-scale spatiotemporal interactions among species [23], and thus may provide a fundamental understanding of the risk of TB transmission at the wildlife/livestock interface. We deployed GPS technology on cattle and wild boar in DNP to test the hypothesis that patterns of resource selection and spatiotemporal overlap between these two species increase the local risk of interspecific disease transmission. Specifically, we aimed to determine where and when the activity patterns of cattle and wild boar overlapped and whether areas with the greatest potential overlap corresponded with areas with high incidence of TB in cattle.

## Material and methods

### Study area

**Figure 1:**
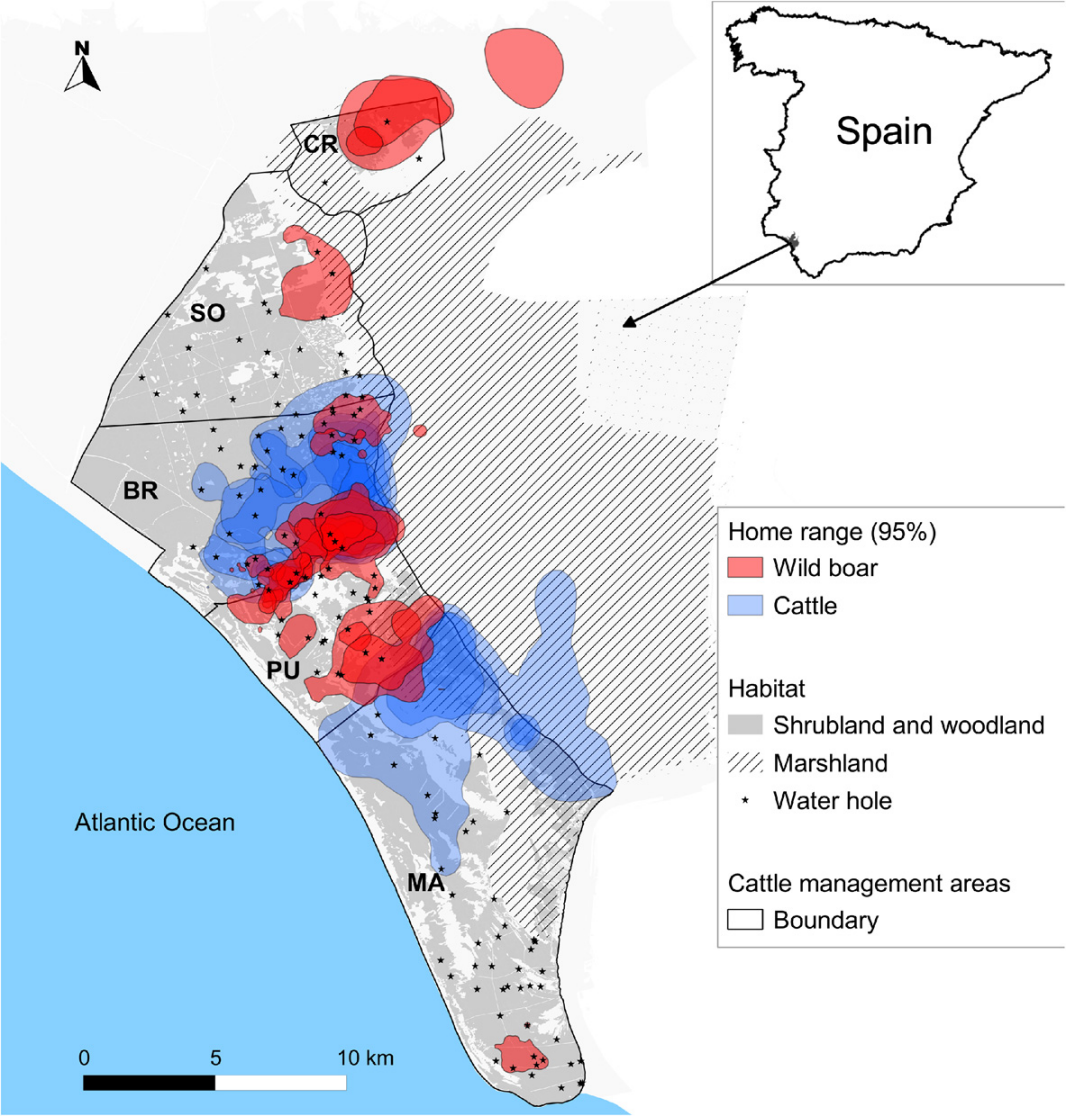
**Study area.** Location of the study area, Doñana National Park (DNP), Huelva province, southern Spain. Home ranges (defined as the 95% isopleth of kernel density estimators) of 18 wild boar and 12 domestic cattle GPS-collared between July 2011 and October 2013 within five cattle management areas are shown.

We conducted the study in DNP (37°0′ N, 6°30′ W), a protected nature reserve located on the Atlantic coast of southern Spain (Figure 1). The region has a Mediterranean climate, classified as dry sub-humid with marked seasons. In the wet season (December–May), marshlands are flooded and ungulates graze in elevated shrublands. Ungulates in DNP are mostly food limited during summer (June–September), when wetlands and natural water bodies dry up causing senescence of herbaceous vegetation. However, a north–south humid ecotone habitat exists year-round between the elevated shrublands and the low dry marshlands; vegetation within this ecotone is dominated by *Scirpus maritimus* and *Galiopalustris sp.* with *Juncus maritimus* associations (see Additional file 1 for further details on habitat types).

The study area has moderate to high densities of red deer, fallow deer and wild boar throughout DNP. A traditional breed of cattle (locally called “marismeña”) is farmed within five cattle management areas in DNP (Figure 1). Coto del Rey (CR) is the northern border of DNP and contains no cattle husbandry. The central area includes three cattle enclosures: SO (*n* = 350 cattle; density = 5.7 cattle/km^2^), BR (*n* = 168 cattle; density = 2.6 cattle/km^2^), and PU (*n* = 152 cattle; density =4.0 cattle/km^2^). Marismillas (MA) is the southern-most area (*n* = 318 cattle; density = 3.1 cattle/km^2^). Each cattle management area is surrounded by a cattle-proof fence, which limits the movements of each herd to within their designated management area. However, social groups (overwhelmingly females) showing individual ranging behaviour may be differentiated within each cattle management area [24]. The incidence of TB in cattle is high within DNP (9.23% per year on average), and TB prevalence in wild boar (45–52%), red deer, and fallow deer (14–19%) populations is also high [21,22]. DNP has been proposed as a natural scenario for describing the epidemiology of shared diseases in wild and domestic ungulates [21,22,25].

### Animal capture and monitoring

We used data from 18 wild boar and 12 head of cattle from the marismeña breed that were equipped with GPS radio-collars between July 2011 and October 2013. Animal capture followed a protocol approved by the Animal Experiment Committee of Castilla-La Mancha University and by the Spanish Ethics Committee, and designed and developed by scientists (B and C animal experimentation categories) in accordance with EC Directive 86/609/EEC for animal handling and experiments. We captured wild boar using six padded foothold cage traps monitored using camera traps (see [26] for further details). Captured wild boar were anaesthetized, weighed, ear tagged, radio-collared and assessed for condition, age and sex. The anaesthetic protocol (3 mg/kg of tiletamine-zolazepam and 0.05 mg/kg of medetomidine) followed Barasona et al. [26]. Of the collared wild boar, 11 were males (3 subadults, < 24 months; 8 adults) and seven were females (2 subadults; 5 adults). All collared cattle were adult females. We captured wild boar in different trapping areas across DNP in order to collar a sample of animals from multiple social groups. Cattle from different social groups were radio-collared during routine veterinary inspections of cattle restrained in the farm’s cattle yards. Although cattle were collared in several cattle management areas, the intense trapping efforts were carried out in BR cattle management area, where both species were concurrently monitored (Figure 1).

Radiocollars were programmed to acquire one GPS location per hour and to transmit accumulated packets of 20 locations using GSM (Microsensory System, Spain) [27]. Data collected included date, time, geographic coordinates, and location acquisition time (LAT, which is a measure of the precision of a fix and ranges between 0–160 s). First, we screened GPS locations with LAT ≥ 154 s to detect anomalous fixes (manufacturer’s technical data; Microsensory System, Spain). Using this criterion, 189 and 66 GPS fixes were considered anomalous and thus removed from wild boar and cattle databases, respectively. We also discarded GPS locations obtained during the day of collar deployment and of collar retrieval to avoid possible anomalous behaviour associated with handling procedures, even though differences in behaviour post-handling were not detected elsewhere [26]. Positional error associated with GPS locations averaged 26.6 m (SD = 23.5 m), based on stationary tests from 19 collars (1637 locations in total) carried out in the centre of our study area (i.e., open sky). Fix-rate success averaged 81.2% and 94.0% for wild boar and cattle, respectively. We explored whether the lower fix-rate success obtained for wild boar introduced habitat-induced biases [28] or dial-induced biases (e.g., due to wild boar using dense vegetation as rest sites during the day [29,30]. However, no significant differences were found in mean LAT values among habitats (Kruskal-Wallis test, z = 48.00, *p* > 0.05) or between day and night (z = −1.88, *p* > 0.05). Consequently, we did not correct for habitat-induced fix-rate bias.

### Coarse-scale spatial overlap between wild boar and cattle

We estimated annual and seasonal (winter, spring, summer and autumn) home-ranges (HR; 95% Utilization Distribution, UD; [31]) and core-areas (CA; 50% UD) used by each collared animal using the fixed-kernel function from the ADEHABITAT package [32] in R version 2.15.2 [33]. Kernels were estimated using the reference bandwidth method [34] because the least-squares cross-validation method failed to converge for six animals with large sample sizes [35]. Fixed-kernel density estimators allow identification of disjunct areas of activity [34], which can be particularly important for assessing interspecific patterns of space use in heterogeneous environments like DNP.

Home ranges and CA for each individual animal were used to estimate annual and seasonal spatial overlap [36] between wild boar and cattle within the BR cattle management area (Figure 1), where both species were concurrently monitored. Spatial overlap was calculated as the area of overlap in HR or CA between wild boar and cattle divided by (1) the total area of HR or CA for wild boar (i.e., overlap for wild boar relative to cattle), or (2) the total area of HR or CA for cattle (i.e., overlap for cattle relative to wild boar).

### Fine-scale spatial interaction between wild boar and cattle

The extent of overlap in HR and CA provides only a coarse indicator of the potential interactions between two species because HR and CA estimators represent only the outline of a distribution of locations [36]. To assess annual and seasonal fine-scale interactions and differences in the use of available resources between cattle and wild boar, we estimated latent selection difference functions (LSDs) [37,38]. The GPS locations were transformed into 26 m radius circular buffers (to account for GPS positional error) [39], and within each buffer we calculated: straight-line distance (km) to nearest artificial water hole (DW); straight-line distance (km) to nearest marsh–shrub ecotone (DE); proportional cover of dense scrub (LT1); proportional cover of low-clear shrubland (LT2); proportional cover of herbaceous grassland (LT3); proportional cover of woodland (LT4); proportional cover of bare land (LT5); and proportional cover of watercourse vegetation (LT6; see Additional file 1). These predictor variables were selected because of their biological relevance for explaining ungulate distribution in DNP (see [40]). Landcover data was obtained from Andalusia Environmental Information [41]. Collinearity among predictor variables was screened using a Spearman’s pairwise correlation coefficient value of |r| > 0.5 [42].

We estimated LSDs using logistic regression [43] and the “RMS” package [44] in R. For this analysis, we coded locations from cattle as 1 and those from wild boar as 0, i.e. we assessed cattle resource selection or avoidance relative to wild boar. In this analysis, landcover variables with significant positive coefficients indicate those most preferred by cattle relative to wild boar, whereas those with significant negative coefficients indicate those most avoided by cattle relative to wild boar. Distance to-variables, however, should be interpreted the opposite way. Variables with non-significant coefficients represent those habitats with the highest potential for interspecific interactions, because there is no difference in the use or selection of these resources between the two species. The results from LSD analyses can then be used to make inferences about the differences or similarities in fine-scale habitat use and spatial overlap between the two species. The main assumption of LSDs is that all resources should be equally available to both species within the study area. To fulfill this assumption, we only used locations from collared animals located in the central cattle management area (BR; 13 wild boar and 10 cattle; Figure 1) that occurred within annual and seasonal inter-species CA (50% UD) overlap contours, i.e. the area where the greatest inter-species interactions could occur. To account for an unbalanced sampling design and non-independence of observations from the same individual, we estimated robust standard errors using the Huber–White sandwich estimator [45], grouping data by individual.

We randomly split the annual and seasonal datasets, using 70% of locations to parameterize the models (training datasets) and the remaining 30% of locations for model validation (validation datasets) [46]. The best annual and seasonal models were obtained using a forwards–backwards stepwise procedure on the training datasets based on Akaike Information Criteria (AIC) [47]. We assessed predictive capacity of the best annual and seasonal models using calibration plots. Calibration plots were constructed by testing the annual or seasonal best models on the corresponding validation dataset, and then plotting the observed and predicted frequency of observations in each of 10 equal-size intervals of predicted probabilities (0–1). A model with high predictive capacity should show perfectly aligned points along a 45° line (see [48]). We also assessed the predictive capacity of each model with the area under the receiver operating characteristic curve (AUC), to rate the ability of the models to correctly discriminate between cattle and wild boar locations. The AUC ranges from 0.5 for models with no discrimination ability to 1 for models with perfect discrimination ability [48].

Annual and seasonal best LSD models were used to spatially map the relative probability of use (*P*) by cattle relative to wild boar. Areas with values of *P* of approx. 0.5 were considered as those where the highest relative probability of spatial interaction between both species could occur [38]. Accordingly, we constructed a spatial interspecific interaction (SII) index for the whole of BR using the rule: SII = (1 – *P*) if *P* ≥ 0.5, and SII = *P* if *P* < 0.5. Further, because models estimating resource use by a species can be used to predict the species’ distribution in other geographical areas (e.g. [49]), we used models trained with data from BR to extrapolate *P* and derived SII index within 1 ha cells across the whole of DNP. Predicted SII values across DNP were correlated with TB epidemiological data (see below).

### Sampling and TB diagnosis

Between 2006 and 2013, 570 wild boar were opportunistically shot by park rangers in DNP, and necropsied as part of the DNP health-monitoring programme (see [21] for details). We recorded the location where animals were shot, the year in which they were sampled, and their TB lesion score, gender and age. Necropsies were performed by qualified wildlife veterinarians that had extensive experience in the diagnosis of macroscopic TB-compatible lesions. Veterinarians performed detailed inspections of the entire animal, including lymph nodes and abdominal and thoracic organs [50]. Cultures using pyruvate-enriched Löwenstein-Jensen medium were performed to confirm TB infection. During the same time period and as part of the TB control programme in DNP, cattle populations within the SO, BR, PU and MA management areas were tested for TB by veterinary authorities using skin tests, and slaughtered if found positive. Prevalence and incidence (as used for cattle because the entire population was tested during annual sanitary campaigns) of TB were estimated for each cattle management area for wild boar and cattle populations, respectively. Finally, we assessed whether there were significant differences in the SII index (annually as well as seasonally) among cattle management areas with high and low TB-incidence in cattle using Mann–Whitney U-tests. All statistical analyses were performed in R version 2.15.2 [33].

## Results

### Interspecific interactions

We collected 44 699 locations from wild boar and 47 213 locations from cattle during the study period. Collared wild boar were distributed across all five cattle management areas, whereas collared cattle were only present in BR and MA (Figure 1). The GPS locations were homogeneously collected throughout the study period for all seasons and for both species (see Additional file 2).

**Figure 2:**
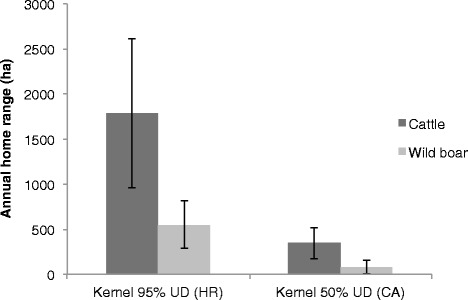
**Comparison of mean annual domestic cattle and wild boar home ranges.** Home range sizes (ha) derived using fixed-kernel density estimators for 95% utilization distribution (UD) and 50% UD. Kernels were estimated using data from 12 cattle and 18 wild boar GPS-collared between July 2011 and October 2013 in Doñana National Park, Spain. Error bars indicate SE.

**Figure 3:**
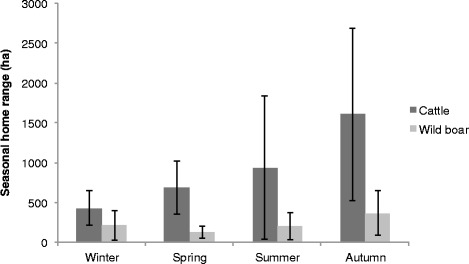
**Comparison of mean seasonal domestic cattle and wild boar home ranges.** Home range sizes (ha) derived using fixed-kernel density estimators for 95% utilization distribution. Kernels were estimated using data from 12 cattle and 18 wild boar GPS-collared between July 2011 and October 2013 in Doñana National Park, Spain. Error bars indicate SE.

There was a stark contrast between estimated annual HR and CA sizes for cattle and wild boar (Figure 2), with cattle using significantly larger areas (average ± SE, HR = 1787.78 ± 826 ha; CA = 346.24 ± 174 ha) than wild boar (HR = 551.33 ± 260 ha; CA = 86 ± 77 ha), (ANOVA, F_1, 28_ = 16.57 for HR; F_1, 28_ = 15.21 for CA; both *p* < 0.001). There were significant seasonal differences in HR sizes for cattle (F_3, 8_ = 3.69, *p* = 0.023), but not for wild boar (F_3, 14_ = 2.47, *p* > 0.05) (Figure 3). The percent overlap in HR and CA between cattle and wild boar varied among seasons, with percent overlap being highest in autumn and lowest in winter (Table 1). Overall, > 60% of wild boar HR overlapped areas used by cattle, whereas ≤ 40% of the HR of cattle overlapped areas used by wild boar. Wild boar CA showed high overlap with areas used by cattle in spring, summer and autumn (66–78% overlap) but not in winter (only 23%).

**Table 1:** **Seasonal and annual coarse-scale overlap**

	**Between-species overlap (%)**				
	**Season**				**Annual**
	**Winter**	**Spring**	**Summer**	**Autumn**	
Wild boar relative to cattle (HR; **CA**)	62.9; **23.0**	85.8; **66.4**	76.4; **70.0**	96.2; **77.7**	96.6; **63.4**
Cattle relative to wild boar (HR; **CA**)	35.7; **9.0**	21.8; **14.6**	21.9; **17.2**	40.1; **24.7**	35.5; **21.2**

Spatial overlap between wild boar and domestic cattle within the BR cattle management area (see Figure 1). Percent overlap was estimated using fixed-kernel density estimators for 95% (HR) and 50% (CA; in bold) utilization distribution. Kernels were estimated using data from 12 cattle and 18 wild boar GPS-collared between July 2011 and October 2013 in Doñana National Park, Spain.

**Figure 4:**
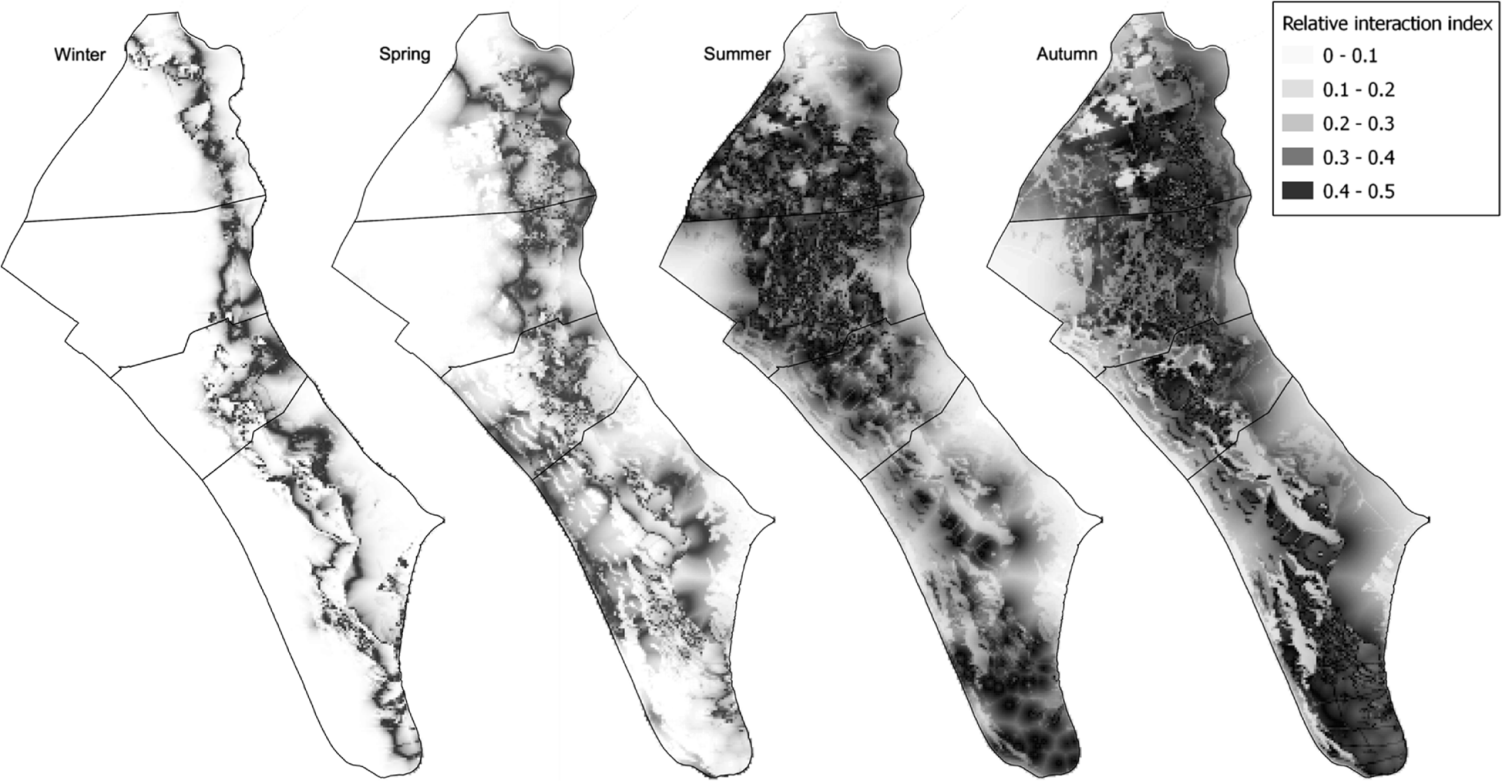
**Pattern of seasonal interspecific interaction.** Spatial gradient in seasonal predicted interspecific interaction index (0 = low interaction, 0.5 = maximum interaction) between wild boar and domestic cattle in Doñana National Park, July 2011–October 2013. Predicted probability of interaction between the two species was derived from four seasonal Latent Selection Difference models (see Table 2).

Fine-scale assessment of spatial interactions between wild boar and cattle revealed that the environmental variables explaining relative habitat selection by cattle and wild boar differed among seasons (Table 2). During winter and spring, cattle used areas significantly further from water sources (DW) than wild boar; however, use of water sources did not differ significantly between the two species during summer and autumn. Cattle and wild boar selection for themarsh–shrub ecotone (DE) did not differ in any of the seasons analyzed. Conversely, cattle showed consistent avoidance of areas with a higher proportion of dense scrub (LT1) relative to wild boar across all seasons. Relative to wild boar, cattle showed avoidance of areas with higher proportions of low-clear shrubland (LT2), herbaceous grassland (LT3) and watercourse vegetation (LT6) during winter and spring. However, during summer and autumn, cattle and wild boar did not differ in their use of these three habitats. Annually, cattle and wild boar did not differ in their selection for areas close to water sources (DW) or for the marsh–shrub ecotone (DE). Validation of LSD models showed that all had good discriminatory power (all AUC > 0.7) and predictive reliability (Additional file 3), supporting their use in extrapolating spatial patterns of SII across the whole of DNP (Figure 4; see also Additional file 4 for annual SII). In general, areas with a high probability of use by both species (high potential interaction) were mostly associated with the marsh-shrub ecotone and permanent water sources (Table 2 and Additional file 4), especially during summer and autumn (dark areas in Figure 4), whereas shrub-woodlands and temporal grass-marshlands had a low probability of interaction.

**Table 2:** **Result of the models**

	**LSD seasonal models**								**LSD annual model**	
	**Winter**		**Spring**		**Summer**		**Autumn**		**AUC = 0.75**	
	**(AUC = 0.83)**		**(AUC = 0.86)**		**(AUC = 0.76)**		**(AUC = 0.71)**			
	**β**	**SE**	**β**	**SE**	**β**	**SE**	**β**	**SE**	**β**	**SE**
Intercept	2.72***	1.608	1.16*	0.481	1.75	1.06	1.11	1.291	2.31*	1.095
DW	2.46*	0.001	2.11*	0.001	1.26 ns	0.001	1.09 ns	0.001	1.46 ns	0.001
DE	−1.46 ns	0.003	−1.26 ns	0.001	−0.79 ns	0.001	0.80 ns	0.001	−1.59 ns	0.001
LT1	−2.90**	0.018	−2.36*	0.009	−3.12**	0.010	−2.19*	0.011	−3.75***	0.009
LT2	−3.37***	0.016	−2.69**	0.007	−0.87 ns	0.012	−1.22 ns	0.012	−3.22**	0.009
LT3	−3.13**	0.017	−3.17**	0.008	−1.18 ns	0.010	−0.88 ns	0.010	−2.25*	0.010
LT4	−1.36 ns	0.015	−4.19***	0.007	−1.46 ns	0.011	−2.12*	0.012	−2.55*	0.009
LT6	−2.95**	0.022	−3.57***	0.008	−1.41 ns	0.012	−1.34 ns	0.011	−3.34***	0.010

Model coefficients (β), standard errors (SE) and area under receiver operating characteristic curve (AUC) from latent selection difference (LSD) functions used for determining relevant factors explaining differences in habitat use by wild boar (coded as 0) and cattle (coded as 1) in Doñana National Park, Spain, July 2011–October 2013. Variable names are described in Additional file 1 and in the methods section. Habitat selection by cattle relative to wild boar was assessed seasonally and annually.

*P*-values are shown as: ns = *p* > 0.05, * = *p* < 0.05, ** = *p* < 0.01, *** = *p* < 0.001.

### TB and spatial overlap

**Figure 5:**
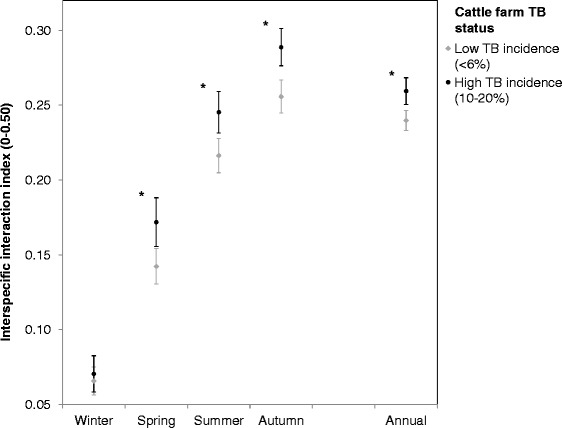
**Relation between spatial interspecific interaction indexes and TB-incidence in cattle.** Differences in the mean predicted value of seasonal and annual spatial interspecific interaction index (0 = low potential interaction, 0.5 = maximum interaction) among cattle management areas with low and high TB-incidence in cattle. Error bars indicate SE. * = significant differences among areas computed from Mann–Whitney U-tests (*p* < 0.05).

Based on culture confirmed lesions, infection was detected in 55.7% (SE = 4.1%; *n* = 570) of wild boar tested. The prevalence of TB in wild boar was 45.9% (SE = 3.8%; *n* = 174) in MA, 64.7% (SE = 5.8%; *n* = 68) in PU, 46.6% (SE = 3.7%; *n* = 174) in BR, 73.9% (SE = 4.6%; *n* = 92) in SO, and 72.6% (SE = 5.7%; *n* = 62) in CR. Official skin testing of 1,139 cattle in DNP from 2006 to 2013 revealed a mean incidence of 9.0% TB reactors (SE = 4.9%). Mean prevalence of TB in wild boar differed significantly among cattle management areas when these areas were grouped into low (MA = 4.1% and BR = 5.6% TB-incidence in cattle; average_wild boar TB-prevalence_ = 46.3%) or high (PU = 18.1% and SO = 11.8% TB-incidence in cattle; average_wild boar TB-prevalence_ = 69.3%) TB-incidence in cattle (F_1, 2_ = 24.96; *p* < 0.05). Interestingly, the mean predicted value of annual SII (fine-scale spatial interspecific interaction) was also significantly higher in high TB-incidence areas than in low TB-incidence areas (Z = 88; *p* < 0.05; Figure 5). These differences were significant in spring, summer and autumn but not in winter (Figure 5).

## Discussion

We assessed fine-scale spatiotemporal interactions between wild and domestic hosts of TB in order to better understand what role resource selection may play in cross-species disease transmission. To our knowledge, this is the first study that has conducted a fine-scale spatial analysis aimed at explaining the patterns of disease transmission at the wild boar/cattle interface. We found that similar use of water resources by cattle and wild boar resulted in high potential interspecific interaction around these landscape features, especially during the dry season. This high spatial overlap at such small spatial extents (e.g. waterholes are only 15 m in diameter) could influence interspecific transmission rates of TB in this Mediterranean system. Our research contributes to an applied understanding of multi-host disease ecology and will help to better target actions and implement control strategies for TB at the wildlife/livestock interface.

We found that cattle had larger HR and CA than wild boar, indicating that these two species have different space use requirements as well as ranging behaviours. Large-scale ranging behaviour in “marismeña” cattle within DNP is mostly determined by human decisions rather than by species-specific traits [24]. Each cattle management area contains a free-ranging cattle herd which is controlled and regulated according to the Cattle Use Plan [51]. The mean HR and CA for wild boar recorded in our study area are somewhat lower than those reported in previous studies in other Mediterranean areas [52,53]. Differences may be related to food availability, population density, activity behaviour and/or composition of social groups [29,53]. Additional research would be required to elucidate which factors regulate ungulate spatial behaviour in DNP. The spatial distribution of cattle (using 95% or 50% UD) overlapped a large proportion of wild boar HR (97%) and CA (63%), and cattle frequently traversed areas used intensively by wild boar. Given that wild boar HR and CA were comparatively smaller than those of cattle, the concentration of important resources and cattle activity within areas commonly used by wild boar created situations that facilitated interaction (or at least spatial overlap) between the two species. Additionally, fine-scale spatial analyses suggest that within areas intensively used by wild boar there was limited spatial interspecific avoidance (also see [19,20]).

Although there were some similarities in the patterns of resource use in all seasons, wild boar and cattle generally used different resources during winter and spring in DNP. Conversely, limited differences in resource selection during summer and autumn resulted in negligible spatial segregation, and thus probably high encounter rates, between the two species during these seasons. This suggests that interspecific contact and subsequent disease transmission between cattle and wild boar is likely to be highest in drier seasons. Interestingly, the spatial distribution of the interspecific interaction index was consistently high at the marsh–shrub ecotone during all seasons. This is probably because this heterogenous habitat offers important resources for ungulates throughout the year, such food and shelter (also see [40]). Furthermore, the predicted increase in spatial overlap and fine-scale interactions between wild boar and cattle during summer and autumn is likely related to the increased use of areas where forage and water are still available, when seasonal drought severely reduces the availability of resources in Mediterranean areas [54].

Characterizing and quantifying the potential interactions and the likelihood of disease transmission between domestic and wild hosts is crucial to understanding the complex dynamics of multi-host systems [11,12]. The results from our assessment of the spatial ecology and interactions of wild boar and cattle suggest that environmental and/or interspecific behavioural factors could favour disease transmission at the livestock/wildlife interface. We found that spatial variation in the incidence of TB in cattle in DNP was positively associated with the prevalence of TB in necropsied wild boar, which is consistent with the hypothesis that TB transmission occurs among ungulates, as has previously been argued from both field and molecular epidemiology [21,22]. In the case of wild boar, the high disease prevalence based on culture (up to 50%) observed in DNP is remarkable and indicative of a high risk of disease transmission [22,55], with about one third of pigs in a random sample expected to be actively excreting mycobacteria by several routes (mainly oro-nasally) [56].

The epidemiological interaction between the two host species described above was further supported by the fact that areas with high TB-incidence in cattle were also the areas with higher predicted spatial interaction between cattle and wild boar. This suggests that the dynamics of disease transmission in DNP are partly driven by the presence of environmental features that facilitate spatiotemporal overlap between hosts, as indicated by LSD models. The humid marsh–shrub ecotone and the surrounding water holes were the habitats with the highest potential interaction between wild boar and cattle. These landscape features may act as potential sources of *M. tuberculosis* complex for the host community [19,20,57]. For instance, Kukielka et al. [19] showed that shared water resources in South Central Spain were risky points where TB transmission could occur by indirect contact. Interestingly, we found that the predicted spatial interspecific interaction was highest in areas with high TB-incidence in cattle (and high TB-prevalence in wild boar) during summer and autumn, i.e. the time of year when species are most water-limited. These complex epidemiological scenarios have also been described in dry areas from Africa where cattle share water holes and diseases with wildlife [58]. In South Spain, a recent study reviewed the environmental persistence of *M. tuberculosis* complex and found that wildlife/livestock interactions occur much more often at water sources than would be expected by chance alone [19]. Aggregation of ungulates is promoted around water points, and this subsequently enhances the opportunities for transmission of diseases [59,60]. This may arise because ungulates come into contact with either a higher proportion of individuals from the same or different species, or with a more heavily contaminated environment (i.e. direct and/or indirect mycobacteria transmission). Spread of TB may occur indirectly from contaminated vegetation, water, mud or fomites [61]. Wild boar activity around these water sources (such as wallowing, brushing, drinking, defecating, urinating, and mating) is likely to result in environmental contamination and TB transmission to other hosts.

We used GPS telemetry data from concurrently monitored domestic cattle and wild boar to describe spatiotemporal interactions by means of new analytical procedures [37,38] and from these inferred associated implications for TB transmission. Within this framework, we considered fine-scale spatial overlap in habitats selected by cattle and wild boar as a proxy of interspecific contact. Although we did not measure contact directly, the difficulty of estimating realistic frequency of contact between species, most of which are predicted to be indirect, has been highlighted previously (e.g. [6,62]). However, recent studies have attempted to measure interspecific contact rates in relation to the dynamics of disease transmission. For example, contact rates have been estimated by direct observation of domestic and wild animals in open habitats where they are easily observed, such as alpine meadows (e.g. [63]). However, this approach was not feasible in our study area because visibility is impeded by closed scrub. Other recent studies using telemetry data have defined critical time and space windows between pairs of GPS locations, and thus only assumed that interspecific contact had occurred within this critical window [62]. Approaches based on proximity loggers potentially have the ability to estimate contact rates between individuals often to within a few meters; however, the performance of these devices is often poor, providing data that is only indicative of contact rates rather than actual contact rates where interactions occur [64]. Further, within an epidemiological context, their utility is constrained to direct rather than indirect disease transmission. The LSD modelling procedure [37] we used proved a reliable tool to estimate annual and seasonal similarities in the use of shared resources, which is valuable for the study of diseases for which direct as well as indirect interactions among sympatric species are of importance in transmission dynamics, as our case [19]. However, the approach was limited in that we could not demonstrate that the spatial overlap between cattle and wild boar occurred within a sufficiently fine-scale temporal window to be directly related to the transmission of TB. Despite this limitation, the LSD approach can provide spatial predictions which can be extrapolated to a larger area where hypotheses related to the spatial risk of interspecific disease transmission can be tested. Additionally, future research could use a combination of proximity loggers and GPS technology to validate rates of interspecific contact, quantify the potential for indirect disease transmission, and identify habitats where both these events occur most frequently.

Epidemiologists and policy makers need to understand the complex interspecific interactions among potential hosts to identify risk factors for disease transmission and prescribe targeted management actions [65]. Our results highlight aspects of the hosts’ ecology and behaviour that are likely to affect the probability of interspecific disease transmission. Further, our results identify factors that need to be considered in order to prevent interactions between wild and domestic ungulates at key disease reservoir sources, such as permanent water sources in ecosystems with marked dry seasons. Although welfare of wild animals must be considered, it may be possible to segregate livestock and wild ungulates in areas surrounding permanent water sources. For example, farm biosecurity measures, like small-scale fencing, exclusion gates or deterrents [66], could be implemented at points such as water sources to prevent wild ungulate access to these areas. Recent innovations in South Spain showed that effective segregation strategies of wild ungulates at water points have the potential to reduce interspecific contact and TB transmission at the wildlife/cattle interface [20]. Furthermore, research is being conducted currently into field vaccination of wild boar against *M. bovis* using oral baits [67]. Ideally, tools from several fields of study should be combined into integrated control plans to minimize pathogen transmission [17] and to improve the cost-effectiveness of strategies such as host population control through random or selective culling or through habitat management [68].

## Additional files

Additional file 1
**Covariates used in the spatial analysis.** Environmental predictors, descriptions, mean values (M) and standard deviations (SD) of GPS locations buffers versus total study area grids used in the analysis of resource separation patterns between cattle and wild boar at Doñana National Park. Additional file 2
**GPS data collection throughout the study period.** Duration of the GPS data collection for each collared wild boar and cattle throughout the study period in Doñana National Park, Spain. Additional file 3
**Calibration plots of the predictive performance of the models.** Assessment of the predictive performance of the best seasonal and annual models (see Table 2). Each plot shows the relationship between the predicted probability to be used by cattle in relation to wild boar and the observed proportion of cattle locations on the validation dataset. Additional file 4
**Pattern of annual interspecific interaction.** Spatial gradient in predicted annual interspecific interaction index (0 = low interaction, 0.5 = maximum interaction) between domestic cattle and wild boar in Doñana National Park, Spain, July 2011–October 2013. Predicted probability of interaction between the two species was derived from an annual Latent Selection Difference model (see Table 2).
