# Supplementary material for: Spatiotemporal interactions between wild boar and cattle: implications for cross-species disease transmission
Source: Vet Res. 2014 Dec 12;45(1):122. doi: 10.1186/s13567-014-0122-7 (PMC4264384; doi:10.1186/s13567-014-0122-7)
Supplement: Additional file 1 — Covariates used in the spatial analysis. Environmental predictors, descriptions, mean values (M) and standard deviations (SD) of GPS locations buffers versus total study area grids used in the analysis of resource separation patterns between cattle and wild boar at Doñana National Park. [file 13567_2014_122_MOESM1_ESM.docx]

| Code | Variable | GPS location buffer M ± SD  (N = 54 886) | Total study area M ± SD  (N = 29 532) |
| --- | --- | --- | --- |
| DW | Distance to nearest water point (km) | 0.49 ± 0.38 | 0.97 ± 0.71 |
| DE | Distance to nearest marsh-shrub ecotone (km) | 1.07 ± 1.23 | 2.33 ± 2.15 |
| LT1 | Dense scrub (%) | 18.42 ± 34.71 | 11.39 ± 26.51 |
| LT2 | Low-clear shrub (%) | 21.63 ± 36.92 | 30.56 ± 39.41 |
| LT3 | Herbaceous grassland (%) | 35.35 ± 42.25 | 11.55 ± 30.78 |
| LT4 | Woodland (%) | 3.58 ± 16.79 | 17.69 ± 34.04 |
| LT5 | Bare land (%) | 2.37 ± 12.59 | 10.64 ± 26.11 |
| LT6 | Watercourse vegetation (%) | 18.44 ± 34.77 | 10.92 ± 27.84 |
