# Supplementary figures and images for: Spatiotemporal interactions between wild boar and cattle: implications for cross-species disease transmission
Source: Vet Res. 2014 Dec 12;45(1):122. doi: 10.1186/s13567-014-0122-7 (PMC4264384; doi:10.1186/s13567-014-0122-7)

**
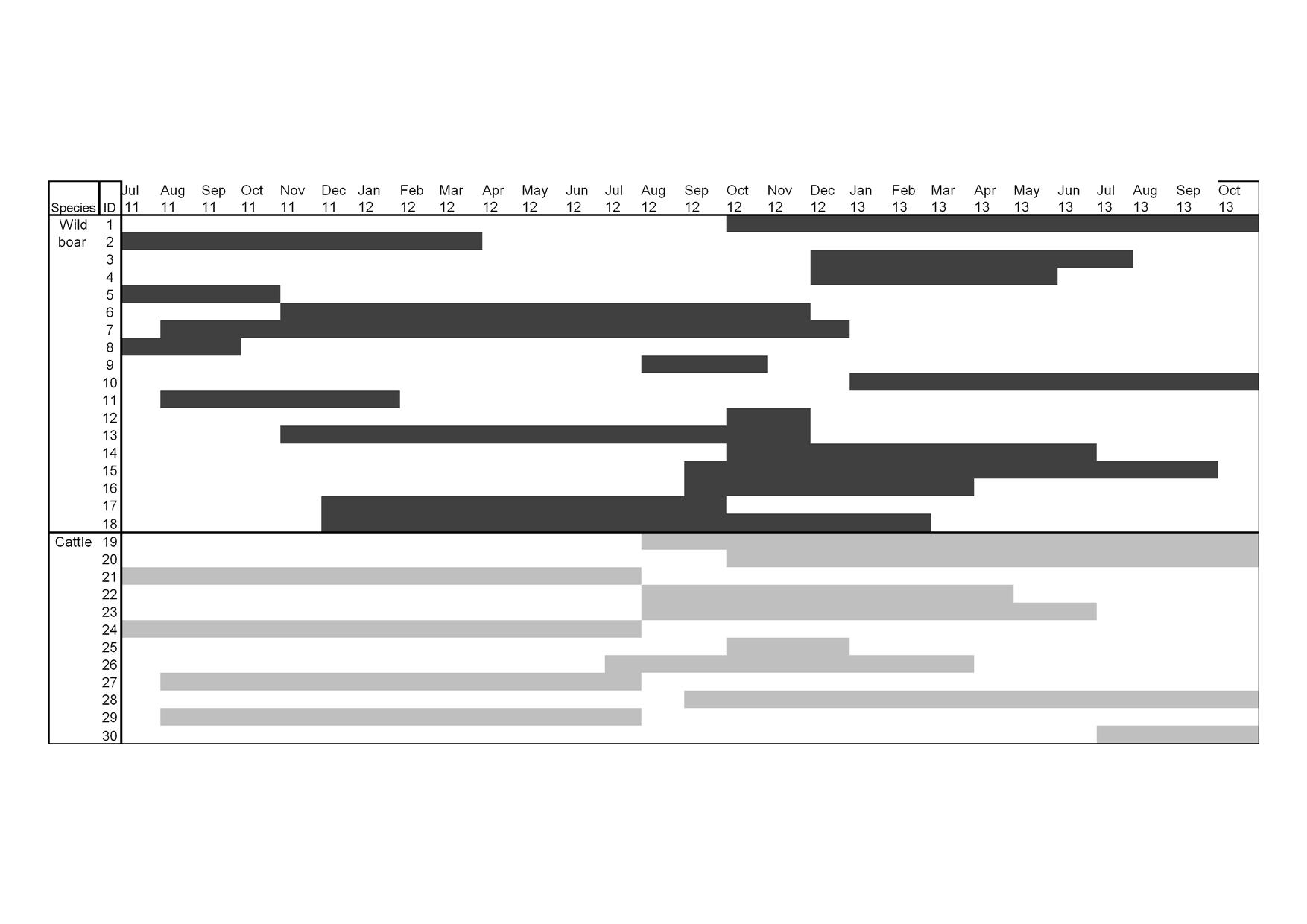
**

Supplement: Additional file 2 — GPS data collection throughout the study period. Duration of the GPS data collection for each collared wild boar and cattle throughout the study period in Doñana National Park, Spain. [file 13567_2014_122_MOESM2_ESM.docx]

**Predicted probability**

**Predicted probability**


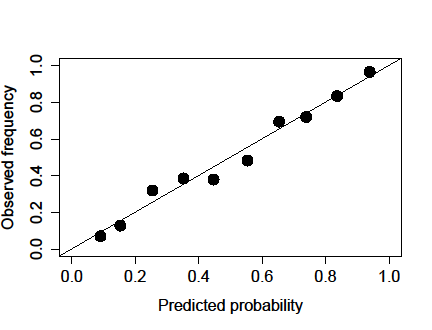

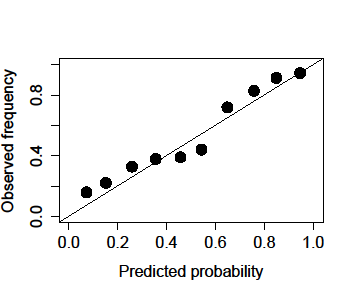

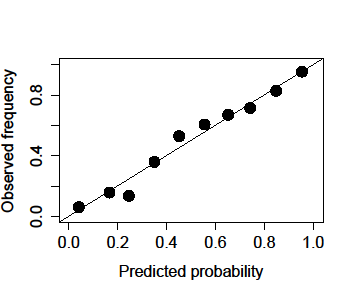

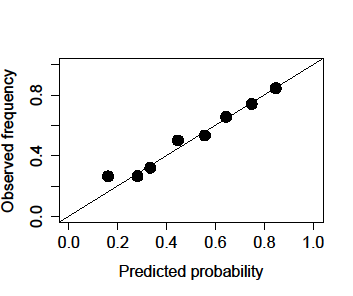

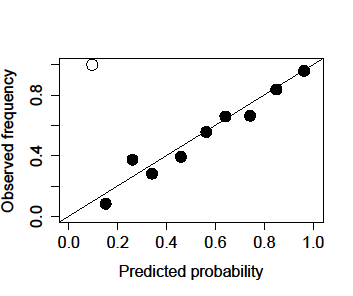


**Winter**

**Spring**

**Summer**

**Autumn**

**Annual**

Supplement: Additional file 3 — Calibration plots of the predictive performance of the models. Assessment of the predictive performance of the best seasonal and annual models (see Table 2). Each plot shows the relationship between the predicted probability to be used by cattle in relation to wild boar and the observed proportion of cattle locations on the validation dataset. [file 13567_2014_122_MOESM3_ESM.docx]

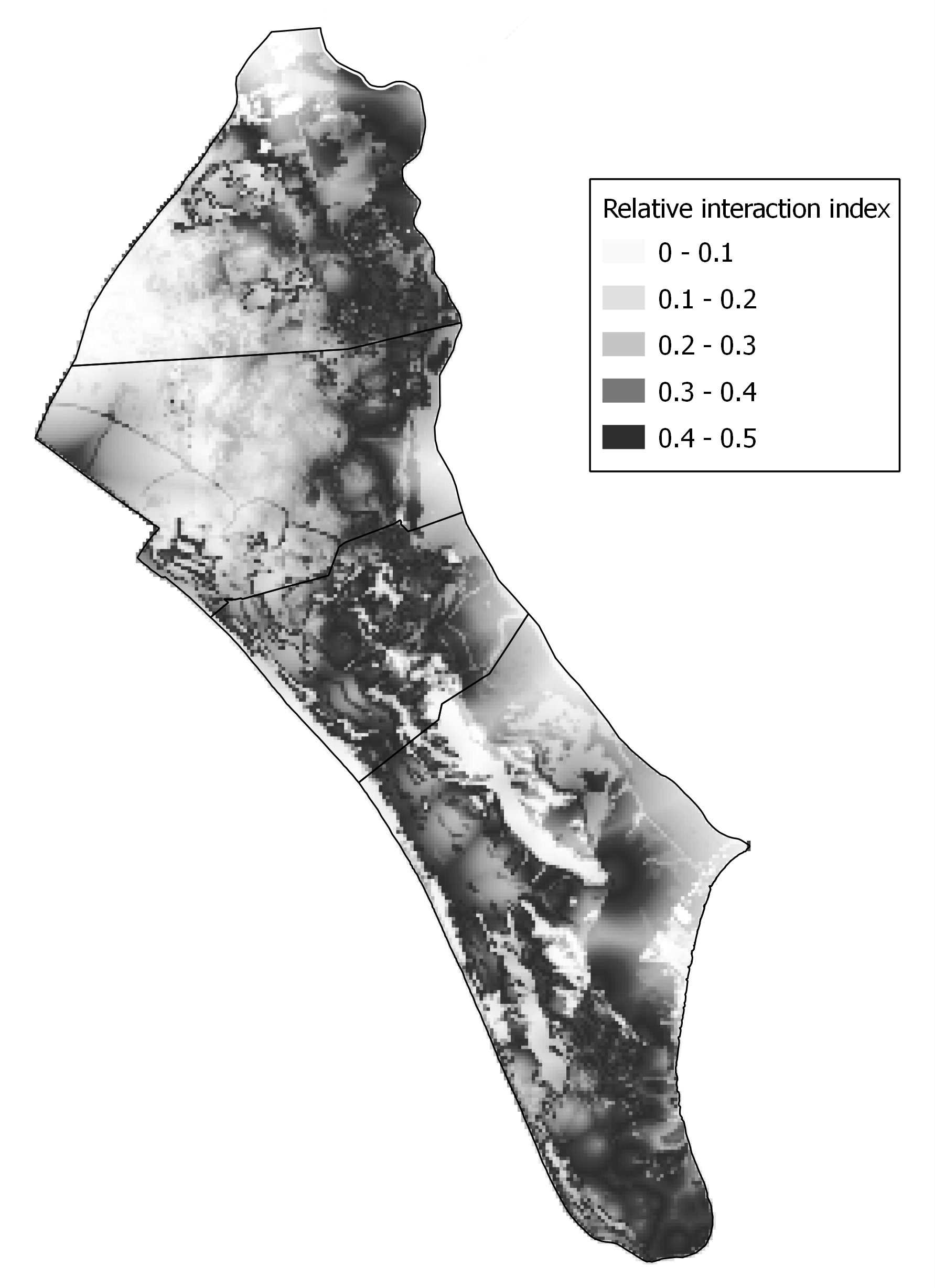

Supplement: Additional file 4 — Pattern of annual interspecific interaction. Spatial gradient in predicted annual interspecific interaction index (0 = low interaction, 0.5 = maximum interaction) between domestic cattle and wild boar in Doñana National Park, Spain, July 2011–October 2013. Predicted probability of interaction between the two species was derived from an annual Latent Selection Difference model (see Table 2). [file 13567_2014_122_MOESM4_ESM.docx]
